# Wavelength-adaptable effective q-plates with passively tunable retardance

**DOI:** 10.1038/s41598-019-48163-8

**Published:** 2019-08-15

**Authors:** Radhakrishna B, Gururaj Kadiri, G Raghavan

**Affiliations:** Theoretical Studies Section, Materials Science Group, Indira Gandhi Centre for Atomic Research, HBNI, Kalpakkam, 603102 India

**Keywords:** Liquid crystals, Optical manipulation and tweezers

## Abstract

Wave retarders having spatially varying optical axes orientations, called q-plates are extremely efficient devices for converting spin to orbital angular momentum of light and for the generation of optical vortices. Most often, these plates are designed for a specific wavelength and have a homogeneous constant retardance. The present work provides a polarimetric approach for overcoming both these limitations. We theoretically propose and experimentally demonstrate q-plates with tunable retardance, employing a combination of only standard q-plates and waveplates. A clear prescription is provided for realizing wavelength indepedent q-plates for a desired retardance, with a potential for ultrafast switching. Apart from the potential commercial value of the proposed devices, our results may find applications in quantum communication protocols, astronomical coronography, angular momentum sorting and in schemes that leverage optical vortices and spin to orbital angular momentum conversion.

## Introduction

Light beams having a heterogeneous distribution of polarization in their transverse plane are called vector beams^1^. Such vector beams have widespread applications in areas ranging from optical tweezing^2^, in achieving sharper focusing^3^ and for the generation of higher-dimensional quantum states of photons for use in quantum key distribution protocols^4,5^. In the last couple of decades, vector beams with a helical wavefront, possessing an optical singularity in their beam axis, called vector-vortex beams or “twisted light”, have been extensively investigated^6^. These beams carry an orbital angular momentum (OAM)^7^ which is to be distinguished from spin angular momentum (SAM) associated with circularly polarized light^8^. Helical beams are generated in a variety of ways: using spiral wave plate^9^, sub-wavelength gratings^10^, cylindrical lenses^11^, holographic patterns^12,13^, spatial light modulators^14^ and plasmonic meta-surfaces^15^.

Another efficient way is through the use of specially engineered liquid crystal based waveplates called q-plates, which have a uniform retardance but with spatially varying optic axis^16^. The advent of q-plates has opened the flood gates of research and applications that exploit the inter play of SAM and OAM^17^. Conventionally, the optic axis orientation, *α*, of q-plates varies linearly with the azimuthal angle, $\varphi$, given by $\alpha (\varphi )=q\varphi +{\alpha }_{0}$. Such q-plates are defined by three parameters $(q,{\rm{\Gamma }},{\alpha }_{0})$ where, *q* is an integer or a half-integer, called the topological charge, ${\rm{\Gamma }}$ represents the retardance, and ${\alpha }_{0}$ the offset angle. Although standard q-plates are predominantly of half-wave retardance, those with a retardance ${\rm{\Gamma }}\ne \pi$ have also found important applications. For instance, using a ${\rm{\Gamma }}=\frac{\pi }{2}$ q-plate, full Poincare beams^18^ have been realized^19^. On the other hand, a circularly polarized light through a q-plate having ${\rm{\Gamma }}=\pi$ (half-wave q-plate hereafter) converts into circularly polarized light of opposite helicity, in addition to picking up an OAM of magnitude $2q\hslash$ per photon. This process is referred to as SAM to OAM conversion (STOC), and has found numerous applications^17,20,21^. For many of these applications, it is important to have a precise control over the fraction of light undergoing STOC. For instance, controlled STOC has been employed for realizing quantum random walks, where the SAM functions as the coin space, while the OAM functions as the walk space^22–25^. In these experiments, the fraction of light undergoing STOC was controlled by tuning the retardance of the q-plates using an externally applied voltage^26^. Retardance of the q-plates can also be tuned by varying the temperature^27^, but this method suffers from a very slow response time. One could also use the intensity of the light itself to control the STOC, but this is a non-linear phenomenon^28–30^.

Retardance of a q-plate depends strongly on the wavelength of the incident light, while its topological charge and off-set angle remain independent of it. Quite often, commercially available standard q-plates exhibit the advertised retardance only at around the design wavelength. Indeed, the performance of a commercial q-plate at wavelengths different from the operational wavelength has been recently studied in^31^. Of late, generation of achromatic optical vortices has been recieving wide interest, owing to their applications primarily in the field of astronomical coronagraphy^32–35^. While different achromatic methods of achieving optical vortices have been proposed^36–39^, achromatic q-plates have received scant attention^40–45^.

Achromaticity and retardance tunability of homogeneous waveplates have been achieved using a sequence of waveplates of appropriate retardances and orientations^46–49^. Motivated by this idea, we seek to design “effective q-plates”, whose retardance is tunable across a broad range of wavelengths, using a combination of q-plates and waveplates. By effective q-plate we mean, the sequence of q-plates is describable as a single q-plate. In other words: (i) The resultant optic axis of the sequence ${\alpha }_{e}(\varphi )$ also varies linearly with the azimuthal angle, ${\alpha }_{e}(\varphi )={q}_{e}\varphi +{\alpha }_{0e}$ and (ii) The resultant retardance of the sequence ${{\rm{\Gamma }}}_{e}$ is independent of $\varphi$.

In this paper, we study the effective q-plates emerging from a sequence of three q-plates. We propose two distinct non-trivial means of realizing these effective q-plates. In each of these cases, we prove that the retardance of the effective q-plates is tunable. We experimentally validate this result by demonstrating fractional STOC, which hitherto has been possible only by varying applied voltage or temperature. In addition, we show that the retardance tunability of these effective q-plates can be extended to a broad range of wavelengths.

## Results

For convenience, we represent a q-plate with parameters $(q,{\rm{\Gamma }},{\alpha }_{0})$ by the notation ${W}_{{\rm{\Gamma }}}(q,{\alpha }_{0})$. This notation offers the flexibility of representing even the homogeneous waveplates (like half-wave and quarter-wave plates), where the orientation of the optic axis remains constant. A homogeneous waveplate with retardance ${\rm{\Gamma }}$ and optic axis orientation $\alpha$ is represented in this notation as ${W}_{{\rm{\Gamma }}}(0,\alpha )$.

Consider three q-plates ${W}_{{{\rm{\Gamma }}}_{1}}({q}_{1},{\alpha }_{01})$, ${W}_{{{\rm{\Gamma }}}_{2}}({q}_{2},{\alpha }_{02})$ and ${W}_{{{\rm{\Gamma }}}_{3}}({q}_{3},{\alpha }_{03})$ arranged in a sequence. It can be seen that if

1 $${{\rm{\Gamma }}}_{1}={{\rm{\Gamma }}}_{3},{q}_{1}={q}_{3}\,{\rm{and}}\,{\alpha }_{01}={\alpha }_{03},$$

then condition (i) of realizing effective q-plates is satisfied, independent of parameters of the central q-plate. The effective retardance ${{\rm{\Gamma }}}_{e}$, when constraints of eq. (1) are satisfied, is

2 $${\textstyle \cos \,\tfrac{{{\rm{\Gamma }}}_{e}}{2}=\,\cos \,\tfrac{{{\rm{\Gamma }}}_{2}}{2}\,\cos \,{{\rm{\Gamma }}}_{1}-\,\sin \,\tfrac{{{\rm{\Gamma }}}_{2}}{2}\,\sin \,{{\rm{\Gamma }}}_{1}\,\cos \,2({\rm{\Delta }}q\varphi +{\rm{\Delta }}{\alpha }_{0})}$$

where ${\rm{\Delta }}q=|{q}_{2}-{q}_{1}|$ and ${\rm{\Delta }}{\alpha }_{0}=|{\alpha }_{02}-{\alpha }_{01}|$ (see Methods for details). Condition (ii), that the effective retardance ${{\rm{\Gamma }}}_{e}$ is independent of azimuthal angle $\varphi$, is satisfied under the following two non-trivial cases:

- Case (a): The outer plates have a retardance ${{\rm{\Gamma }}}_{1}$ equal to odd-multiple of *π*.
- Case (b): The central and outer q-plates have identical topological charge, ${\rm{\Delta }}q=0$.

**Figure 1:**
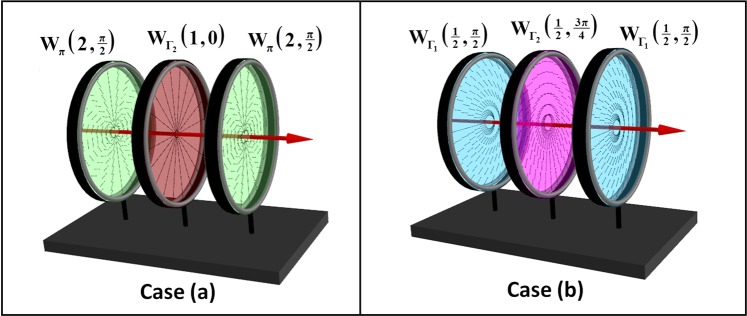
Schematic of the two non-trivial cases, where a sequence of three q-plates acts like a single effective q-plate. In both the cases, the outer q-plates have identical retardance and are also oriented identically. In case (**a**), the outer plates are half-wave q-plates while the central plate is a q-plate of arbitrary retardance. In case (**b**), all three q-plates have identical topological charge.

These two cases are depicted in Fig. 1.

### Case (a): Effective q-plates realized using half-wave q-plates

Consider first the arrangement as shown in Fig. 1a. A q-plate ${W}_{{{\rm{\Gamma }}}_{2}}({q}_{2},{\alpha }_{02})$ is placed in between two identically oriented half-wave q-plates ${W}_{\pi }({q}_{1},{\alpha }_{01})$. The effective retardance ${{\rm{\Gamma }}}_{e}$, the effective topological charge ${q}_{e}$ and effective off-set angle ${\alpha }_{0e}$ in this case are given by (see Methods for details):

3 $$\begin{array}{rcl}{{\rm{\Gamma }}}_{e} & = & {{\rm{\Gamma }}}_{2},\\ {q}_{e} & = & 2{q}_{1}-{q}_{2},\\ {\alpha }_{0e} & = & 2{\alpha }_{01}-{\alpha }_{02}.\end{array}$$

In short,

4 $${W}_{\pi }({q}_{1},{\alpha }_{01})\to {W}_{{{\rm{\Gamma }}}_{2}}({q}_{2},{\alpha }_{02})\to {W}_{\pi }({q}_{1},{\alpha }_{01})\equiv {W}_{{{\rm{\Gamma }}}_{2}}(2{q}_{1}-{q}_{2},\,2{\alpha }_{01}-{\alpha }_{02})$$

The effective topological charge is ${q}_{e}=2{q}_{1}-{q}_{2}$. This arrangement can therefore be used for synthesizing q-plates of different topological charges. For instance, using commercially available q-plates of topological charge 1 and 0.5, it is possible to realize an effective q-plate with topological charge ${q}_{e}=1.5$. Similarly, placing ${W}_{{\rm{\Gamma }}}({q}_{2},{\alpha }_{0})$ between two homogeneous half-wave plates ${W}_{\pi }(0,0)$ yields an effective q-plate ${W}_{{\rm{\Gamma }}}(-{q}_{2},-\,{\alpha }_{0})$, where the topological charge is inverted. These results, but restricted only to half-wave q-plates have been reported in^50–52^. The current analysis generalizes them to arbitrary retardance q-plates.

The offset angle of the q-plates, in general, can be altered by merely rotating them. The only exception to this is q-plates with $q=1$, owing to their rotational symmetry of optic axis^16^. Hitherto, experiments involving ${W}_{{\rm{\Gamma }}}(1,{\alpha }_{0})$ q-plates necessitated fabricating distinct q-plates for each ${\alpha }_{0}$^53^. This requirement can be mitigated by realizing an effective ${W}_{{\rm{\Gamma }}}(1,{\alpha }_{0})$ plate. One way towards this is through ${W}_{\pi }(0.5,0)\to {W}_{{\rm{\Gamma }}}(0,-\,\alpha )\to {W}_{\pi }(0.5,0)$. This yields an effective q-plate ${W}_{{\rm{\Gamma }}}(1,\alpha )$ (see eq. (4)), the offset angle of which can be changed by merely rotating the central homogeneous waveplate ${W}_{{\rm{\Gamma }}}(0,-\,\alpha )$.

From eq. (4), the retardance of the effective q-plate is equal to the retardance of the central q-plate. This result holds good even if the central plate is homogeneous (${q}_{2}=0$). It is therefore possible to realize a q-plate of any retardance, by sandwiching a homogeneous waveplate of that retardance between two half-wave q-plates. Further, this result can be utilized for realizing a tunable retardance q-plate ${{\rm{\Gamma }}}_{e}\in (0,2\pi )$, provided retardance of the central homogeneous waveplate is itself tunable, ${{\rm{\Gamma }}}_{2}\in (0,2\pi )$. Retardance tunable homogeneous waveplates have been realized in many ways^46,47,54–56^. By replacing the central plate of Fig. 1a with any such tunable retarder yields an effective q-plate with tunable retardance.

As an illustration, here we demonstrate tunable retardance q-plate, by sandwiching a Berek plate^55^ between two $q=1$ half-wave q-plates. Berek plate was set for five different retardances, ${\rm{\Gamma }}\in (0,\frac{\pi }{4},\frac{\pi }{2},\frac{3\pi }{4},\pi )$ while its orientation ${\alpha }_{02}$ was fixed at 0. The performance of this arrangement is validated against that of the ${W}_{{\rm{\Gamma }}}(2,0)$ q-plate, for plane and circularly polarized light beams.

**Figure 2:**
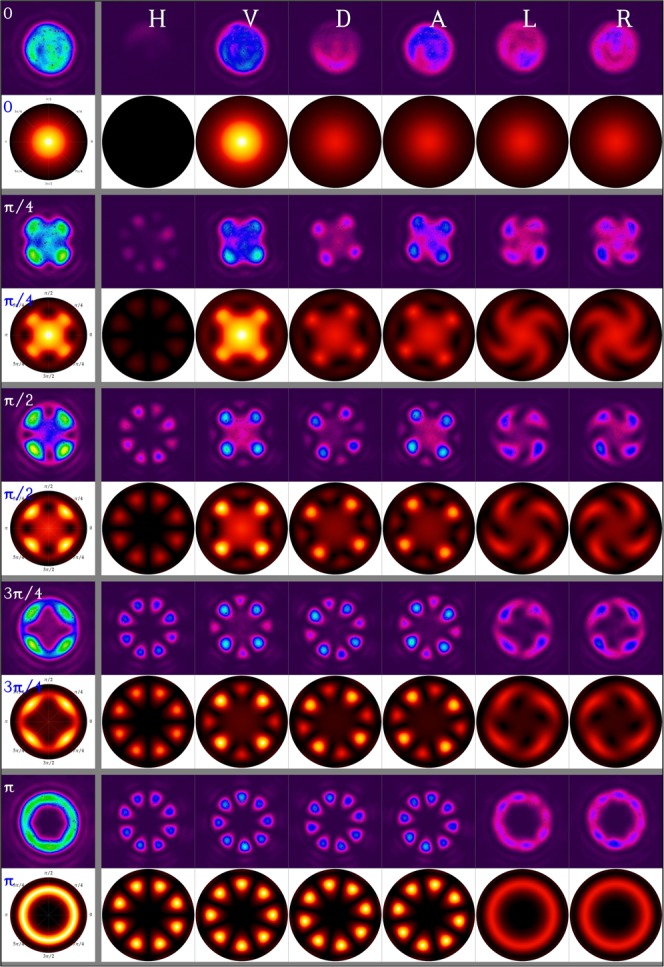
Experimental and theoretical intensity profiles (unprojected and six cardinal projections) of a vertically polarized light beam evolving through an effective q-plate tuned for five different retardances.

At every retardance of the Berek plate, the vertically polarized light beam is sent through the aforementioned arrangement and the intensity profile in the transverse plane of the evolved vector beam is recorded. Further, to validate the polarization distribution, the evolved beam was projected into six cardinal directions, horizontal (H), vertical(V), diagonal (D), antidiagonal (A), left circular (L) and right circular (R). The theoretical intensity profiles obtained when a vertically polarized light beam exits a ${W}_{{\rm{\Gamma }}}(2,0)$ q-plate, set for the same retardances, and evolves for the same distance as in the experiment, is computed using the formulae derived in references^19,57^. The experimental and the corresponding theoretical images are shown in Fig. 2.

We observe eight lobes in the plane polarization projections intensity profiles, indicating an effective topological charge ${q}_{e}=2$, which is double that of the individual q-plates, as expected from eq. (4). These lobes are identical and of equal intensity for ${{\rm{\Gamma }}}_{e}=\pi$. Further, the unprojected intensity at the beam center gradually falls with increasing retardance ${{\rm{\Gamma }}}_{e}$, completely vanishing when the retardance is ${{\rm{\Gamma }}}_{e}=\pi$. Further, since the input beam is vertically polarized, the intensity profile in its orthogonal projection, that is along the horizontal component is always a doughnut with singularity at the center, for any retardance. At ${{\rm{\Gamma }}}_{e}=\pi$, the horizontal and vertical projections are identical and have equal power. The excellent match between the intensity profile obtained with our gadget and the theoretically expected profile confirms the suitability of our setup for obtaining q-plates with tunable retardance.

A major application of tunable retardance q-plates has been in obtaining a controlled spin to orbital conversion of angular momentum of light. Light beam having a definite spin (polarization) and orbital angular momentum sent through a q-plate of retardance ${\rm{\Gamma }}\ne \pi$, gets converted into a superposed beam having no definite spin or orbital angular momentum. Denoting the left (right) circularly polarized beam with OAM of $m\hslash$ units by $|L,m\rangle$ $(|R,m\rangle )$, the action of a q-plate ${W}_{{\rm{\Gamma }}}(q,{\alpha }_{0})$ is given by:

5 $$\begin{array}{rcl}{W}_{{\rm{\Gamma }}}(q,{\alpha }_{0})|L,m\rangle  & = & \cos (\frac{{\rm{\Gamma }}}{2})|L,m\rangle +i\,{e}^{2i{\alpha }_{0}}\,\sin (\frac{{\rm{\Gamma }}}{2})|R,m+2q\rangle ,\\ {W}_{{\rm{\Gamma }}}(q,{\alpha }_{0})|R,m\rangle  & = & \cos (\frac{{\rm{\Gamma }}}{2})|R,m\rangle +i\,{e}^{-2i{\alpha }_{0}}\,\sin (\frac{{\rm{\Gamma }}}{2})|L,m-2q\rangle \end{array}$$

The STOC fraction is obtained by measuring the power in the left and right circular projections. Fraction of the beam that remains in the initial state $|L,0\rangle$ is proportional to ${\cos }^{2}(\frac{{\rm{\Gamma }}}{2})$, while the fraction getting converted to the $|R,2\rangle$ state is proportional to ${\sin }^{2}(\frac{{\rm{\Gamma }}}{2})$. Complete spin to angular momentum conversion is possible only when ${\rm{\Gamma }}=\pi$.

**Figure 3:**
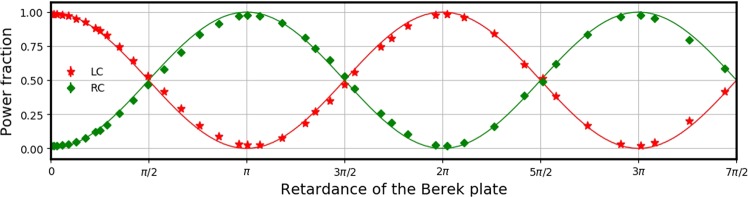
Fraction of power in the left circular (LC) and right circular (RC) projections, plotted as a function of q-plate retardance. The effective q-plate is realized as in Fig. 1a, with the central plate being the Berek plate.

Here we demonstrate STOC using the tunable ${W}_{{\rm{\Gamma }}}(2,0)$ q-plate discussed above, using left circularly polarized Gaussian beam $|L,0\rangle$. Figure 3 shows the power fraction in the left and right circular projections, as a function of the retardance of the central Berek plate (and hence the effective retardance of the q-plate).

In Fig. 3, stars and diamonds indicate the experimentally measured average power fractions in left and right circular projections respectively, at different retardances of the central Berek plate. At every retardance, averaging is done over 30 readings, with each reading lasting for 1 second. The average power fractions are plotted together with their error-bars. Error-bars are the maximum and minimum values of these 30 readings, which happen to be less than 0.1% at every data point, hence too small (smaller than the symbols) to be visible. The continuous lines are the theoretical expectations obtained from eq. (5). Power fraction in left and right circular projections show sinusoidal variation with the retardance of Berek plate, becoming equal at odd multiples of $\frac{\pi }{2}$. The close agreement between theory and experiments demonstrates the excellent functionality of this gadget as a q-plate with tunable retardance.

### Case (b): Effective q-plate realized by using three q-plates of identical topological charge

Here, we analyze case (b) depicted in Fig. 1b, where the condition for obtaining the effective q-plate with uniform retardance is ${\rm{\Delta }}q=0$: all three q-plates have identical topological charge. From eq. (2), the effective retardance ${{\rm{\Gamma }}}_{e}$ in this case is decided by ${\rm{\Delta }}{\alpha }_{0}$, being $2{{\rm{\Gamma }}}_{1}+{{\rm{\Gamma }}}_{2}$ for ${\rm{\Delta }}{\alpha }_{0}=0$ and $2{{\rm{\Gamma }}}_{1}-{{\rm{\Gamma }}}_{2}$ for ${\rm{\Delta }}{\alpha }_{0}=\,\frac{\pi }{2}\,$. The full span of $(0,2\pi )$ retardance is possible using this arrangement when $2{{\rm{\Gamma }}}_{1}-{{\rm{\Gamma }}}_{2}=0$ and $2{{\rm{\Gamma }}}_{1}+{{\rm{\Gamma }}}_{2}=2\pi$, that is when ${{\rm{\Gamma }}}_{1}=\frac{\pi }{2}$ and ${{\rm{\Gamma }}}_{2}=\pi$. The effective retardance ${{\rm{\Gamma }}}_{e}$ and effective orientation ${\alpha }_{0e}$ in this case becomes:

$$\begin{array}{rcl}{{\rm{\Gamma }}}_{e} & = & 4({\alpha }_{02}-{\alpha }_{01}),\\ {\alpha }_{0e} & = & {\alpha }_{01}-\frac{\pi }{4}.\end{array}$$

In our notation,

6 $${W}_{\frac{\pi }{2}}(q,{\alpha }_{01})\to {W}_{\pi }(q,{\alpha }_{02})\to {W}_{\frac{\pi }{2}}(q,{\alpha }_{01})\equiv {W}_{4({\alpha }_{02}-{\alpha }_{01})}(q,{\alpha }_{01}-\frac{\pi }{4})$$

Recall that ${\rm{\Delta }}{\alpha }_{0}=|{\alpha }_{02}-{\alpha }_{01}|$ is relative orientation of the optical axes of outer and central plates at the zero azimuth. Since for q-plates with $q\ne 1$, it is possible to change the off-set angle ${\alpha }_{0}$ by changing their orientation, the effective retardance can be tuned just by rotating either the central or the outer q-plates. It is hence possible to construct a q-plate of tunable effective retardance ${\rm{\Gamma }}\in (0,2\pi )$ using two quarter-wave q-plates and a half-wave q-plate, all having identical topological charge ($\ne 1$). This is yet another way of realizing q-plate with tunable retardance involving three q-plates.

For q-plates with different retardances, ${{\rm{\Gamma }}}_{1}(\ne \frac{\pi }{2})$ and ${{\rm{\Gamma }}}_{2}(\,\ne \,\pi )$, it may not be possible to achieve the complete span of effective retardance ${{\rm{\Gamma }}}_{e}\in (0,2\pi )$. We briefly explore this scenario further in this section. It follows from eq. (2) that a retardance ${{\rm{\Gamma }}}_{e}$ is realizable, by a suitable ${\rm{\Delta }}{\alpha }_{0}$, provided the following condition is satisfied:

7 $$|\frac{\cos \,\frac{{{\rm{\Gamma }}}_{2}}{2}\,\cos \,{{\rm{\Gamma }}}_{1}-\,\cos \,\frac{{{\rm{\Gamma }}}_{e}}{2}}{\sin \,\frac{{{\rm{\Gamma }}}_{2}}{2}\,\sin \,{{\rm{\Gamma }}}_{1}}|\le 1$$

**Figure 4:**
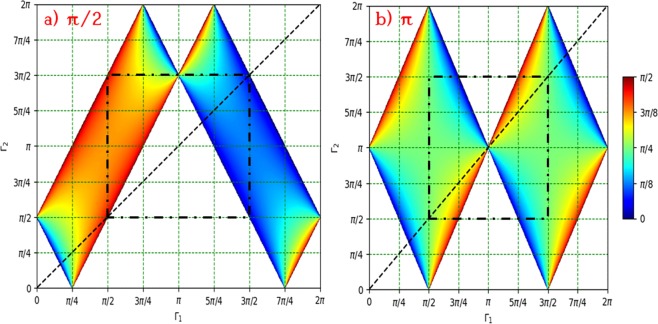
Consider the sequence of q-plates arranged as in case (**b**). Let the outer q-plates be of identical retardance ${{\rm{\Gamma }}}_{1}$ and the central q-plate be ${{\rm{\Gamma }}}_{2}$ (see Fig. 1b). The graph shows the possibility of realizing an effective retardance (**a**) ${{\rm{\Gamma }}}_{e}=\frac{\pi }{2}$ and (**b**) ${{\rm{\Gamma }}}_{e}=\pi$ at different values of $({{\rm{\Gamma }}}_{1},{{\rm{\Gamma }}}_{2})$. The dotted line across the diagonal describes the equation ${{\rm{\Gamma }}}_{1}={{\rm{\Gamma }}}_{2}$ and the color coding depicts relative off-set angle ${\rm{\Delta }}{\alpha }_{0}$ at which these effective retardances are realized.

Figure 4 shows the possible values of ${{\rm{\Gamma }}}_{1}$ and ${{\rm{\Gamma }}}_{2}$ using which an effective retardance of (a) ${{\rm{\Gamma }}}_{e}=\frac{\pi }{2}$ and (b) ${{\rm{\Gamma }}}_{e}=\pi$ can be realized. The color coding is the value of ${\rm{\Delta }}{\alpha }_{0}$ for realizing them. For instance, it is possible to realize ${{\rm{\Gamma }}}_{e}=\frac{\pi }{2}$ with outer q-plates having ${{\rm{\Gamma }}}_{1}=\frac{\pi }{4}$ and the central q-plate having ${{\rm{\Gamma }}}_{2}=\frac{3\pi }{4}$, while it is not possible to achieve this effective retardance with ${{\rm{\Gamma }}}_{1}=\frac{3\pi }{4}$ and ${{\rm{\Gamma }}}_{2}=\frac{\pi }{4}$.

**Figure 5:**
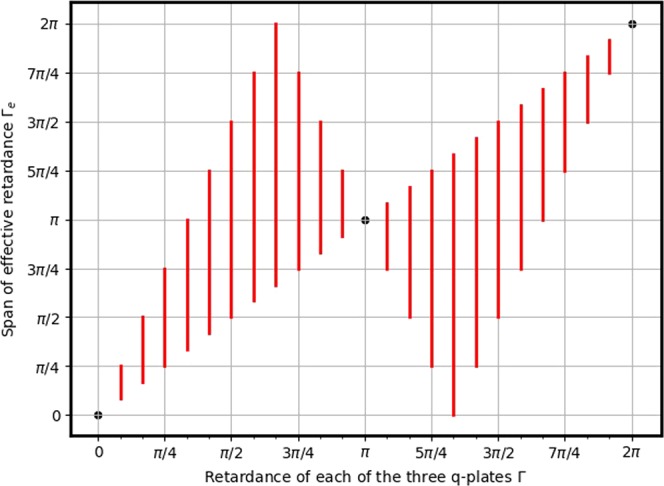
The span of effective retardance ${{\rm{\Gamma }}}_{e}$ realizable from three identical q-plates of retardance ${\rm{\Gamma }}$ arranged as in Fig. 1b.

It would be interesting to know whether it is possible to obtain a desired retardance ${{\rm{\Gamma }}}_{e}$ using three q-plates with identical retardances. The dotted line in the figure is ${{\rm{\Gamma }}}_{1}={{\rm{\Gamma }}}_{2}$ and its intersection with the colored region indicates whether the effective retardance ${{\rm{\Gamma }}}_{e}$ can be realized with three waveplates of identical retardance. For instance, since the point $(\frac{\pi }{4},\frac{\pi }{4})$ lies outside of the colored region in Fig. 4b, it is not possible to realize ${{\rm{\Gamma }}}_{e}=\pi$ using three q-plates with ${{\rm{\Gamma }}}_{1}={{\rm{\Gamma }}}_{2}=\frac{\pi }{4}$. On the other hand, $(\frac{\pi }{2},\frac{\pi }{2})$ lies within the colored region, and hence it is possible to achieve ${{\rm{\Gamma }}}_{e}=\pi$ with ${{\rm{\Gamma }}}_{1}={{\rm{\Gamma }}}_{2}=\frac{\pi }{2}$. Figure 5 shows the span of effective retardance ${{\rm{\Gamma }}}_{e}$ achievable using three identical q-plates having retardance ${\rm{\Gamma }}$. This is computed from eq. (2), by setting ${{\rm{\Gamma }}}_{1}={{\rm{\Gamma }}}_{2}={\rm{\Gamma }}$, ${\rm{\Delta }}q=0$ and by varying ${\rm{\Delta }}{\alpha }_{0}$ from 0 to $\frac{\pi }{2}$. From the plot, it is evident that for ${\rm{\Gamma }} < \pi$, the minimum ${{\rm{\Gamma }}}_{e}$ is ${\rm{\Gamma }}$, while for ${\rm{\Gamma }} > \pi$ the maximum possible ${{\rm{\Gamma }}}_{e}$ is ${\rm{\Gamma }}$. With ${\rm{\Gamma }}=\pi$, the only possible effective retardance is ${{\rm{\Gamma }}}_{e}=\pi$.

While in case(a) the retardance tunability was achieved by replacing the central q-plate by a tunable homogeneous waveplate, in case(b) the retardance tunability is achieved by merely rotating the central q-plate. The possible span of realizable effective retardance in the latter case depends upon the retardances of the outer and the central plates. In the extreme case, the effective q-plate realized using three half-wave q-plates is just another half-wave q-plate, offering zero tunability. Since standard commercial q-plates are often of half-wave retardance, this arrangement may appear to be of little practical use towards realizing tunable retardance q-plates. However, this sequence of three half-wave q-plates will be useful for realizing wavelength-adaptable q-plates, by which we mean an effective q-plate which can be tuned to exhibit any desired retardance at any desired wavelength.

### Realization of wavelength-adaptable q-plates

We now examine the possibility of realizing wavelength-adaptability of q-plates in two cases, (a) and (b). In case (a), for a wavelength different from the operating wavelength of the q-plates, the outer two q-plates have a retardance ${{\rm{\Gamma }}}_{1}$ different from $\pi$, and hence from eq. (2), the effective retardance becomes a function of the azimuthal angle. So the three-plate arrangement of case (a) fails to act like an effective q-plate at wavelengths different from the operating one.

In case (b), on the other hand, the condition for realizing an effective q-plate is ${\rm{\Delta }}q=0$, which is a wavelength-independent constraint. An effective q-plate ${W}_{{{\rm{\Gamma }}}_{e}}({q}_{e},{\alpha }_{0e})$ at one wavelength continues to remain an effective q-plate at a different wavelength, albeit with a different ${{\rm{\Gamma }}}_{e}$ and ${\alpha }_{0e}$. So we explore the possibility of wavelength-independence in this case. For simplicity, consider three identical q-plates having half-wave retardance at a design wavelength ${\lambda }_{d}$: ${\rm{\Gamma }}({\lambda }_{d})=\pi$. At a wavelength $\lambda$ different from ${\lambda }_{d}$, the retardance ${\rm{\Gamma }}(\lambda )$ of each plate is different from $\pi$. From Fig. 5, it is seen that an effective retardance of ${{\rm{\Gamma }}}_{e}=\pi$ is achievable for any retardance ${\rm{\Gamma }}$ within the window $[\frac{\pi }{3},\frac{5\pi }{3}]$. Hence, at any wavelength $\lambda$ such that $\frac{\pi }{3} < {\rm{\Gamma }}(\lambda ) < \frac{5\pi }{3}$, it is possible to realize an effective retardance of $\pi$, using the arrangement of Fig. 1b. Similarly, an effective retardance of ${{\rm{\Gamma }}}_{e}=\frac{\pi }{2}$ is achievable for ${\rm{\Gamma }}$ within $[\frac{\pi }{6},\frac{\pi }{2}]$ or $[\frac{7\pi }{6},\frac{3\pi }{2}]$. So at any wavelengths $\lambda$ such that $\frac{\pi }{6} < {\rm{\Gamma }}(\lambda ) < \frac{\pi }{2}$ or $\frac{7\pi }{6} < {\rm{\Gamma }}(\lambda ) < \frac{3\pi }{2}$, it is possible to realize a retardance of $\frac{\pi }{2}$ using the arrangement of Fig. 1b.

**Figure 6:**
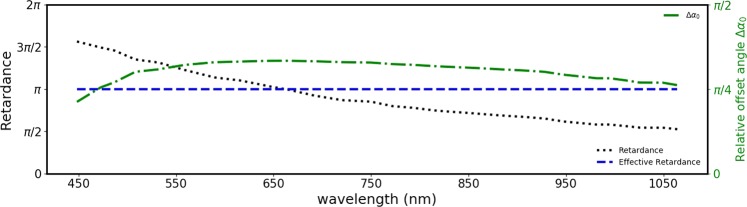
The retardance (dotted line) of a commercial half-wave q-plate (Thorlabs WPV10-633) as a function of wavelength, taken from^31^. Using three of these q-plates, it is possible to realize a q-plate of effective retardance *π* (dashed line) in this wavelength range. The dot-dash line indicates the offset orientation of the central waveplate for achieving this.

For concreteness of this idea, we discuss here the possibility of realizing an effective q-plate having retardance of ${{\rm{\Gamma }}}_{e}=\pi$ over a range of wavelengths, using three standard commercial q-plates (Thorlabs, WPV10-633, with q = 1) designed to provide a retardance ${\rm{\Gamma }}=\pi$ at 633 nm. The ${\rm{\Gamma }}(\lambda )$ plot for these q-plates has been reported recently^31^. While this study was carried out for $q=1$ q-plates, we proceed with the reasonable assumption that the similar dependence holds true even for $q\ne 1$ q-plates. There it was observed that ${\rm{\Gamma }}(\lambda )$ varies inversely with *λ*, with ${\rm{\Gamma }}(450\,{\rm{nm}})=\frac{3\pi }{2}$ and ${\rm{\Gamma }}(1050\,{\rm{nm}})=\frac{\pi }{2}$. Since the retardance variation of this q-plate is confined between $\frac{\pi }{2}$ and $\frac{3\pi }{2}$, which is within the $[\frac{\pi }{3},\frac{5\pi }{3}]$ window, an effective retardance of ${{\rm{\Gamma }}}_{e}={\rm{\pi }}$ can be realized at any wavelength within the range $[450\,{\rm{nm}},1050\,{\rm{nm}}]$. Figure 6 shows the value of ${\alpha }_{02}$, at which the central q-plate needs to be oriented, at every wavelength *λ* to obtain an effective retardance of *π*.

**Figure 7:**
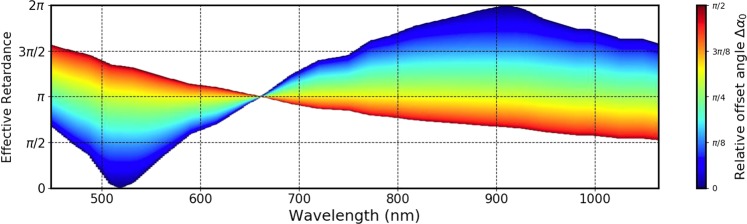
The possibilities of obtaining a retardance as a function of wavelength using three standard half-wave q-plates designed for 633 nm is depicted. The color coding indicates the relative off-set angle ${\rm{\Delta }}{\alpha }_{0}$.

Figure 7 consolidates the possible retardances realizable at different wavelengths, using three Thorlabs WPV10-633 q-plates. The color-coding is for the value of ${\rm{\Delta }}{\alpha }_{0}$. It is evident that a full wavelength-independence is possible only for half-wave retardance ${{\rm{\Gamma }}}_{e}=\pi$. For other retardances, only piecewise wavelength-independence is achievable.

The Fig. 7 can also be used for knowing the possible values of retardances realizable at every wavelength using three of the above q-plates. For instance, while the retardance of *π* is realizable at every wavelength, close to the design wavelength it is only possible to realize an effective retardance of *π*.

## Discussion

To summarize, an exhaustive study of the conditions under which a combination of three q-plates function as an effective q-plate has been carried out. The central result of this work is the identification of two inequivalent configurations for obtaining effective q-plates. First configuration restricts the outer q-plates to be of half-wave retardance, while the second configuration requires the three q-plates to be of identical topological charge. We have shown that it is possible to tune the retardance of the effective q-plate realized by either means, former method being less restrictive than the latter.

As an experimental demonstration towards this, we have realized a retardance tunable q-plate by sandwiching a Berek plate between two half-wave q-plates. The generated vector-vortex beams and the fractional STOC effect are found to compare excellently with theory, validating the practical utility of our method in realizing tunable retardance q-plates. As against the current state of the art, the retardance tunability is achieved here through passive means. This retardance tunability can also be realized using only off-the-shelf q-plates and waveplates, by replacing the Berek plates with a gadget involving a half-wave plate sandwiched between two identically oriented quarter-wave plates. On the other hand, by replacing the Berek plate with a fast acting Pockels cell, one could easily achieve sub-microsecond switching times whereas conventional voltage or temperature tunable q-plates have switching time in the order of milliseconds.

The effective q-plates realized in the second configuration, while utilizing only standard wavelength sensitive q-plates, are shown to function over a wide gamut of wavelengths.

While we have restricted our attention to standard q-plates, our results are general and are applicable even to the so called meta-q-plates^58^. We believe that the retardance tunability, control of topological charge and wavelength-independence offered by our method will be of substantial commercial value towards realizing quantum protocols like quantum key distribution, quantum random walks and will lead to further exploration of optical phenomena involving interplay of SAM and OAM degrees of freedom.

## Methods

The action of a waveplate with retardance ${\rm{\Gamma }}$ and optic axis oriented at an angle $\alpha$ is described by the Jones Matrix ${U}_{{\rm{\Gamma }}}(\alpha )$^59^:

8 $${U}_{{\rm{\Gamma }}}(\alpha )=[\begin{array}{cc}\cos \,\frac{{\rm{\Gamma }}}{2}+i\,\sin \,\frac{{\rm{\Gamma }}}{2}\,\cos \,2\alpha  & i\,\sin \,\frac{{\rm{\Gamma }}}{2}\,\sin \,2\alpha \\ i\,\sin \,\frac{{\rm{\Gamma }}}{2}\,\sin \,2\alpha  & \cos \,\frac{{\rm{\Gamma }}}{2}-i\,\sin \,\frac{{\rm{\Gamma }}}{2}\,\cos \,2\alpha \end{array}]$$

The diagonal elements of this matrix are complex conjugates, while the off-diagonal elements are purely imaginary and equal to each other. The resulting action due to a sequence of waveplates is obtained by the product of the Jones matrix of each of them. The off-diagonal elements of the resulting product matrix need not be purely imaginary. We term a sequence of waveplates as an “effective waveplate”, provided the off-diagonal elements of the product matrix are purely imaginary, as in eq. (8).

Consider three waveplates having retardance ${{\rm{\Gamma }}}_{j}$ and orientated at an angle ${\alpha }_{j},j=1,2,3$. The resultant matrices ${U}_{{{\rm{\Gamma }}}_{1}}({\alpha }_{1}){U}_{{{\rm{\Gamma }}}_{2}}({\alpha }_{2}){U}_{{{\rm{\Gamma }}}_{3}}({\alpha }_{3})$ represents an effective waveplate if ${{\rm{\Gamma }}}_{1}={{\rm{\Gamma }}}_{3}$ and ${\alpha }_{1}={\alpha }_{3}$^46^. The effective retardance ${{\rm{\Gamma }}}_{e}$ and effective orientation ${\alpha }_{e}$ of this effective waveplate are obtained by:

9 $$\begin{array}{rcl}2\,\cos \,\frac{{{\rm{\Gamma }}}_{e}}{2} & = & {\rm{Tr}}[{U}_{{{\rm{\Gamma }}}_{1}}({\alpha }_{1}){U}_{{{\rm{\Gamma }}}_{2}}({\alpha }_{2}){U}_{{{\rm{\Gamma }}}_{1}}({\alpha }_{1})],\\ 2\,\sin \,\frac{{{\rm{\Gamma }}}_{e}}{2}\,\cos \,2{\alpha }_{e} & = & {\rm{Tr}}[-i{U}_{{{\rm{\Gamma }}}_{1}}({\alpha }_{1}){U}_{{{\rm{\Gamma }}}_{2}}({\alpha }_{2}){U}_{{{\rm{\Gamma }}}_{3}}({\alpha }_{3}){\sigma }_{x}]\end{array}$$

where *σ*_*x*_ is the Pauli spin matrix $$[\begin{array}{ll}1 & 0\\ 0 & -\,1\end{array}]$$.

For a q-plate, ${W}_{{\rm{\Gamma }}}(q,{\alpha }_{0})$, the Jones matrix is same as eq. (8), with $\alpha =q\varphi +{\alpha }_{0}$. The condition ${\alpha }_{{}_{1}}={\alpha }_{3}$ translates to ${q}_{1}={q}_{3}$ and ${\alpha }_{01}={\alpha }_{03}$.

### Experimental details

The experiments are carried out using q-plates of Thorlabs make (model number: WPV10-633, with q = 1). The Berek plate is of Newport make (model number: 5540). A He-Ne laser of JDSU make (model number: 1145P) has been employed. Polarized light beam from this source is then expanded to a diameter of about 4 mm, from a combination of achromatic doublet lenses. For carrying out circular projections, achromatic quarter-wave plates of Thorlabs make have been used. Linear polarizer used is of Thorlabs make, which has an extinction ratio of 100,000:1 for the light of 633 nm wavelength. The intensity profile in the transverse plane is captured at a distance of 1 m using a beam profiler of Newport make (model number: LBP2-HR-VIS). The optical power in the STOC experiments is measured using a photodiode sensor of Ophir make (model number PD300-UV).
